# Associations of psychological distress, gaming motives and internet gaming disorder in adolescents: a network analysis

**DOI:** 10.3389/fpsyt.2026.1787380

**Published:** 2026-04-24

**Authors:** Ruiyi Chen, Xiaoyan Peng, Ling Zhao, Hangping Li, Genghua Yao, Hongyan Xu, Lijun Su, Hong Luo, Yong-guang Wang

**Affiliations:** 1 Affiliated Mental Health Center & Hangzhou Seventh People’s Hospital, Zhejiang University School of Medicine, Hangzhou, Zhejiang, China; 2 The Third People’s Hospital of Fuyang District, Hangzhou, Zhejiang, China; 3 Fuyang Education Science Research Office, Hangzhou, Zhejiang, China; 4 Zhejiang Key Laboratory of Drug Prevention and Control Technology, Hangzhou, Zhejiang, China; 5 Qinghai Third People’s Hospital, Xingning, Qinghai, China; 6 School of Mental Health and Psychological Sciences, Anhui Medical University, Hefei, Anhui, China; 7 Zhejiang Provincial Institute of Drug Abuse Research, Hangzhou, Zhejiang, China

**Keywords:** anxiety, depression, gaming motive, IGD, network analysis, stress

## Abstract

**Background and objective:**

The rapid popularization of the Internet among Chinese adolescents has resulted in the emergence of a public major concern known as Internet Gaming Disorder (IGD). As demonstrated by previous studies, an association has been demonstrated among emotional distress, gaming motives and IGD. Nevertheless, the specific pathways connecting these constructs remain to be elucidated. The present study aims to explore the network structure characterizing the interactions among these three constructs and to identify potential targets for psychological interventions.

**Methods:**

This was a cross-sectional survey conducted in city of Hangzhou. A total of 3,795 middle school students were included in the analysis. The 21-item Depression Anxiety Stress Scale (DASS-21), the Motives for Online Gaming Questionnaire (MOGQ), and the Chinese version of the Ten-Item Internet Gaming Disorder Test (IGDT-10) were used to assess emotional distress, gaming motives and IGD symptoms, respectively. Network analyses were performed using R4.5.1 software to explore the interrelationships among emotional distress, gaming motives and IGD symptoms, and identify the core symptoms and bridge symptoms.

**Results:**

In the depression combined network model, the presence of bridge symptoms was indicated by no initiative (D2), gaming for escape or mood relief (IGD8) and fantasy motive (fan). In anxiety combined network model, the bridge symptoms included coping motive(cop), gaming for escape or mood relief (IGD8), withdrawal (IGD2), mouth dryness (A1), and fear of embarrassment (A4). The bridge symptoms in the stress combined network model were gaming for escape or mood relief (IGD8), difficulty winding down (S1), withdrawal (IGD2), nervous energy expenditure (S3), and coping motive (cop).

**Conclusion:**

The present study explored complex network structure among psychological distress, gaming motivation, and IGD. and suggested fantasy and coping motive as bridges connecting psychological distress and IGD. Besides, our research identified no initiative, mouth dryness, difficulty winding down, fear of embarrassment, and nervous energy expenditure as the best targets for intervention to reduce IGD.

## 1 Introduction

According to the 54^nd^ Statistical Report on China’s Internet Development (1), the number of mobile internet users in China reached 1.1 billion as of June 2024. Furthermore, the 5^th^ National Survey Report on Minor’s Internet Usage indicates that the number of minor internet users in China has grown for five consecutive years since 2018, reaching 193 million by 2022 and resulting in an internet penetration rate of 97.2% among minors (2). Consequently, in the context of pervasive mobile internet adoption, internet usage among Chinese minors has attained near-universal levels, signifying its profound integration into their daily lives and learning environments.

In the context of the widespread integration of the internet among minors, online gaming has emerged as a predominant and time-consuming form of leisure, profoundly impacting adolescent daily routines. For individuals who engage extensively with online gaming, the potential for Internet Gaming Disorder(IGD) to emerge as a significant concern is a salient issue (3). A meta-analysis (4) indicates that the average global prevalence of IGD is approximately 3.3%, while the prevalence among children and adolescents has reached as 6.6%. And the prevalence rate of IGD for Chinese samples has been reported ranging from 2.3% (5) to 18% (6). IGD, characterized by persistent gaming leading to clinical impairment, can result in severe health, academic, and social consequences (7). However, it is important to note that the development of this disorder cannot be attributed solely to access, but rather, it is fundamentally linked to intrinsic individual factors.

Motivation is widely regarded as the foundation of most human actions (8), and serves as a key psychological factor in the development of addictive behaviors (9). A substantial body of research has indicated a significant correlation between specific psychological motives and online gaming addiction (10–12). Individuals with gaming addiction frequently experience unmet psychological needs, which can result in a persistent urge to engage in online gaming as a means of fulfilling these needs. For instance, it is well established that escapism motivation has a strong association with IGD (9, 13). Concurrently, IGD has been demonstrates substantial comorbidity with emotional problems (14–16). Numerous studies have shown a strong positive correlation between IGD symptoms and depression in adolescents (17–19). Furthermore, problematic internet use has been found to significantly associated with elevated suicidal ideation (20). On the other hand, anxiety also plays a significant role in IGD. Individuals with high levels of anxiety may resort to internet game as a maladaptive coping mechanism to avoid the stress and fear inherent in real social interactions (21). Individuals diagnosed with anxiety disorders have been shown to exhibit a heightened tendency towards problematic smartphone use and dependency on online gaming (22).

It is imperative to acknowledge that these factors do not function in isolation, but rather interact in a dynamic manner. As demonstrated in the relevant literature, emotional distress, such as depression and anxiety, has been shown to be associated with maladaptive emotion regulation strategies (23). This, in turn, has been demonstrated to function as the primary proximal catalyst for excessive gaming behavior (24). In this pathway, motives frequently function as a pivotal mediating factor, translating underlying emotional vulnerabilities into overt symptoms of IGD (25). Recent empirical evidence supports this model, demonstrating that anxiety and depression significantly mediate the relationship between an individual’s motivational orientation (e.g., controlled causality orientations) and IGD severit^y^ (26). Furthermore, escapist motivation itself is a well-established predictor of negative psychological outcomes, including depression (27). Consequently, the development of IGD can be conceptualized as the synergistic outcome of this interplay, whereby heightened negative emotional states potentiate specific, need-fulfilling gaming motivations, which then directly fuel disordered use.

To unravel such complex interrelationship, network analysis is one of the promising and powerful statistical methods. Traditional latent variable models, such as Structural Equation Modeling (SEM), typically regard symptoms as interchangeable indicators of a latent construct, and thus do not explicitly address the unique importance of individual symptoms or the interactions among them (28). In contrast, network analysis conceptualizes psychological variables as complex systems comprising mutually reinforcing symptoms (29). In this framework, symptoms (conceptualized as nodes) directly interact with and activate one another (forming edges), which better reflects the fine-grained, symptom-level interactions. Furthermore, network analysis can not only identify core nodes within a single network, but also identify bridge nodes between symptom clusters(i.e, specific nodes of a variable can activate another variable) (30). These bridge nodes often represent potential targets for psychological intervention (31). Several network analysis studies have found that specific symptoms such as “withdrawal”, “escape”, “down-hearted”, and “breath difficulty” usually serve as the core link connecting IGD with depression and anxiety (32–34). There have also been studies exploring how distinct gaming motives differentially relate to IGD severity (13, 35). Despite these advancements, most existing network studies have examined these constructs in isolation, or have only focused on pairwise associations(e.g., depression and IGD), a comprehensive investigation of emotional distress, gaming motives, and IGD symptoms interact within a unified system remains unclear. The objectives are twofold: (a) to explore the network structure connecting these three constructs, and (b) to identify the bridge nodes linking emotional distress(depression, anxiety, stress), specific gaming motives, and IGD symptoms. The identification of such bridge nodes could inform the development of targeted psychological interventions.

## 2 Materials and methods

### 2.1 Participants and procedure

This survey was conducted as part of routine mental health assessments among five middle school’s students from the city of Hangzhou in November 2024. A total of 5,191 middle school students completed the online assessments. Following the exclusion of questionnaires with a response time of less than five minutes, as well as those from participants who reported never playing internet games (n = 817), the final analysis was conducted on data from 3,795 participants. The demographic information concerning the participants is displayed in **Table 1**. The present study was approved by the Ethics Committee of the Third People’s Hospital of Fuyang District, Hangzhou (Ref. 2023-2-002-01).

**Table 1:** Demographic characteristics of the sample (n = 3795).

Variables	Mean (SD), Range, %
Age (years old)	15.74 ± 1.53, 2-20
Male gender	1906 (50.2%)
Female gender	1889 (49.8%)
Educational level	
junior high school students	717(18.9%)
senior high school students	3078(81.1%)
IGD scale	3.43(2.98)
Depression scale	8.14(8.05)
Anxiety scale	7.13(7.65)
Stress scale	6.25(7.49)
Internet gaming motives scale	2.65(0.83)

### 2.2 Measures

#### 2.2.1 Internet gaming disorder

Internet gaming disorder symptoms were assessed by the Chinese version of the Ten-Item Internet Gaming Disorder Test (IGDT-10) (36), which is based on the DSM-5 diagnostic criteria of IGD. The IGDT-10 employed a 3-point Likert scale ranging from 0 (‘never’) to 2 (‘often’) (37). With the exception of Criterion 9 (‘Risks or loses an important relationship, job, or training/career opportunity because of playing’), each criterion is evaluated using one item. The Chinese version was previously validated in a Chinese sample, and demonstrated good internal consistency in the current study. The internal reliability for the IGDT-10 instrument was found to be satisfactory in the present study (Cronbach’*ɑ* = 0.88).

#### 2.2.2 Depression, anxiety, stress

The present study utilized the Chinese version of the 21-item Depression Anxiety Stress Scale (DASS-21). The scale consists of three sub-scales, which respectively reflect depressive, anxious, and stressful symptoms. Each sub-scale comprises 7 items, with a rating scale ranging from 0 (not applicable at all) to 3 (applied to a significant extent). The reliability and validity of the scale have been demonstrated in previous research (38). The internal reliability for the DASS-21 in the present study was found to be satisfactory (for total scale, Cronbach’s *ɑ* = 0.96; for sub-scale of depression, Cronbach’s *ɑ* = 0.87;for sub-scale of anxiety, Cronbach’s *ɑ* = 0.88; and for sub-scale of stress subscales, Cronbach’s *ɑ* = 0.88; respectively).

#### 2.2.3 Gaming motives

The Motives for Online Gaming Questionnaire (MOGQ) (39) was utilized to assess gaming motivations in this study. The questionnaire comprised 27 items and was composed of seven domain-specific factors (i.e., Escape, Coping, Fantasy, Skill Development, Recreation, Competition, and Social). Each factor consisted with three to four items. Participants were asked to rate each item on a 5-point Likert scale ranging from 1 (Strongly Disagree) to 5 (Strongly Agree), with higher scores indicating stronger expressions for each gaming motive. In the present study, the internal consistency of the MOGQ was determined to be Cronbach’s *ɑ* = 0.96.

### 2.3 Data analysis

Descriptive statistics were conducted for demographic variables using SPSS27.0, and the scores of the scales and each factor were presented in **Table 2**. As there remains considerable controversy surrounding the optimal approach for the modeling of three-category items in network analysis (40), the present work drew upon extant research. The DASS items were thus dichotomized as ‘0’ and ‘1’ to denote absence (corresponding to the original item value of ‘0’) or presence of symptoms (item value of ‘1’, ‘2’, or ‘3’). In order to align with the dichotomous structure of IGD symptoms as described in the DSM-5, the responses ‘sometimes’ and ‘often’ were recoded as 1. The scores for item 9 and item 10 were then combined. Therefore, in the event that either item 9 or item 10 (or both) are answered as ‘sometimes/often’, a single point will be awarded. As outlined in the previous study (41), the mean value of each type of motive was calculated for the analyses.

**Table 2:** Description, Mean, and SD for network nodes and overall scales.

Node name	Item content/overall scales	Mean	SD
IGD1	Preoccupation	0.64	0.48
IGD2	Withdrawal	0.28	0.45
IGD3	Tolerance	0.35	0.48
IGD4	Loss of control	0.39	0.49
IGD5	Loss of non-gaming interest	0.22	0.42
IGD6	Gaming despite harms	0.33	0.47
IGD7	Deception of others about gaming	0.27	0.45
IGD8	Gaming for escape or mood relief	0.57	0.50
IGD9	Conflict due to gaming	0.38	0.49
D1	No positive	0.41	0.49
D2	No Initiative	0.40	0.49
D3	No look forward	0.41	0.49
D4	Down-hearted	0.42	0.49
D5	Not enthusiastic	0.35	0.48
D6	Worthless	0.21	0.40
D7	Meaningless	0.23	0.42
A1	Mouth dryness	0.43	0.50
A2	Breathing difficulty	0.33	0.47
A3	Trembling sensation	0.31	0.46
A4	Fear of embarrassment	0.62	0.48
A5	Near panic feeling	0.33	0.47
A6	Heart awareness	0.32	0.47
A7	Irrational fear	0.32	0.47
S1	Difficulty winding down	0.51	0.50
S2	Over-reactivity	0.34	0.48
S3	Nervous energy expenditure	0.50	0.50
S4	Agitation	0.45	0.50
S5	Difficulty relaxing	0.43	0.50
S6	Intolerance of interruption	0.47	0.50
S7	Irritability	0.47	0.50
esc	escape	2.49	0.91
cop	coping	3.15	1.06
fan	fantasy	2.27	0.99
ski	skill development	2.71	0.99
rec	recreation	3.28	1.07
com	competition	2.47	1.02
soc	social	2.71	1.02

IGDT and DASS nodes were coded as 0 or 1. IGD, internet gaming disorder, D, depression, A, anxiety; S, stress; esc, escape; fan, fantasy; ski, skill development; rec, recreation; com, competition; soc, social.

Rstudio version 4.5.1 was used for network analysis in the present study. The network was estimated using a mixed graphical model (MGM) in the R package ‘mgm’ (42), and the network structure was visualized in the qgraph package (43). In network analysis, each gaming motive, IGD symptom and emotion symptom (depression, anxiety, stress) was regarded as a ‘node’, and the ‘edges’ between nodes represented partial correlations after statistically eliminating interference from all other nodes (44). Three combined network models were constructed respectively. In Model 1, nodes represented variables of the depression sub-scale of DASS-21, seven dimensions of MOGQ, and IGD symptoms. In the Model 2 and Model 3, the depression sub-scale of DASS-21 was replaced by the anxiety sub-scale and the stress sub-scale respectively. Within all models, blue edges indicate positive relations, and red edges denote negative relations. The hypothesis that wider edges mean stronger associations. In order to reduce the likelihood of spurious or false positive outcomes, the MGM model employs the eLASSO (Enhanced Least Absolute Shrinkage and Selection Operator) method (45) to enhance the interpretability of sparse networks. The selection of the model was conducted using the Extended Bayesian Information Criterion (EBIC).

In the present study, both Expected Influence (EI) and Bridge Expected Influence (BEI) were estimated. EI serves as a crucial metric for assessing network centrality, enabling the identification of the most influential nodes within the network (46). EI is distinguished from other centrality indices by virtue of the fact that it accounts for the sign of edges (positive or negative). This enables it to assess the cumulative influence of nodes more effectively. A higher EI value is indicative of a stronger influence, signifying the greater importance of the node within the overall network. BEI is the centrality indicator of the nodes in the combined network. This is the sum of edge weights connecting a specific node to all other nodes in different symptom communities. In the combined network, a high BEI value indicates a strong influence of one community on others, or vice versa. Bridge nodes were selected using the 80th percentile of BEI values as the cutoff (31). We utilized the networktools package (47) to evaluate the bridge centrality of the nodes.

To test the network robustness and stability, the bootnet package was applied to estimate the network and to conduct statistical analyses and visualizations of centrality. Firstly, based on 1000 non-parametric bootstrap samples, the edge weights and the 95% confidence intervals (95%CI) of the nodes were calculated, and the differences between edge weights or nodes were tested with a significance level of ɑ = 0.05. Secondly, we used case-dropping bootstraps (n = 1000) to estimate the stability of centrality indices for each network model, which calculated the correlation stability coefficient (CS-coefficient) for the EI and BEI indices. The CS-coefficient (48) indicated the proportion of cases that can be dropped from analyses while preserving a correlation of at least r = 0.70, within a 95% confidence interval, and A CS-coefficient should not be below 0.25 and 0.5 indicates good network stability.

## 3 Results

### 3.1 Demographic characteristics and descriptive statistics

**Table 1** shows the demographic characteristics of the sample. **Table 2** summarizes the details of each node of DASS-21, IGDT-10, MOGQ scale.

### 3.2 Network analysis of emotion symptom, internet gaming motives and IGD

**Figure 1:**
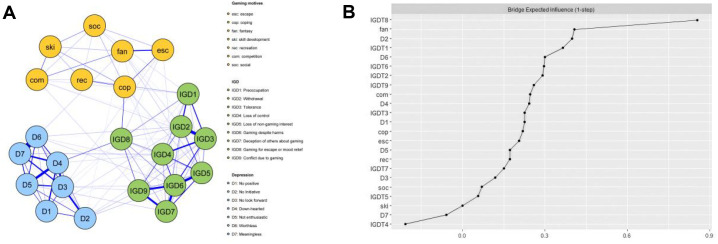
The depression-gaming motives-IGD network model and bridge expected influence (BEI). **(A)** The depression-gaming motives-IGD network model. **(B)** The bridge expected influence indices of the nodes in the network (raw score).

The depression-gaming motives-IGD network (Model 1) is displayed in **Figure 1A**. It is clear that there was a maximum of 253 edges across the communities in the network; in the present research, there were 107 cross-community edges (mean weight= 0.096). Bootstrapped confidence intervals(CIs) of the bridge edge weights are shown in **Supplementary Figure 1**.

**Figure 2:**
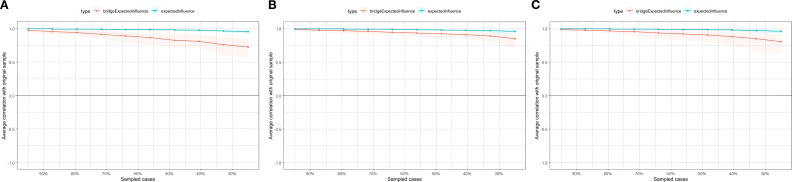
The CS-coefficient of combined network. **(A)** The depression-gaming motives-IGD network model. **(B)** The anxiety-gaming motives-IGD network model. **(C)** The stress-gaming motives-IGD network model.

**Figure 1B** show the bridge expected influence (BEI) values for each node. Gaming for escape or mood relief(IGD8), Fantasy(fan), No initiative(D2) had the largest 1-step value and emerged as bridge nodes in the combined network model, with BEI values of 0.85, 0.41 and 0.40 respectively. As **Figure 2A** shows, the average correlation of BEI indices of the subsample and the original sample clearly demonstrated a downward trend as the subsample size decreased. The CS-coefficient on the BEI was 0.594, which was larger than 0.5, indicating adequate stability.

**Figure 3:**
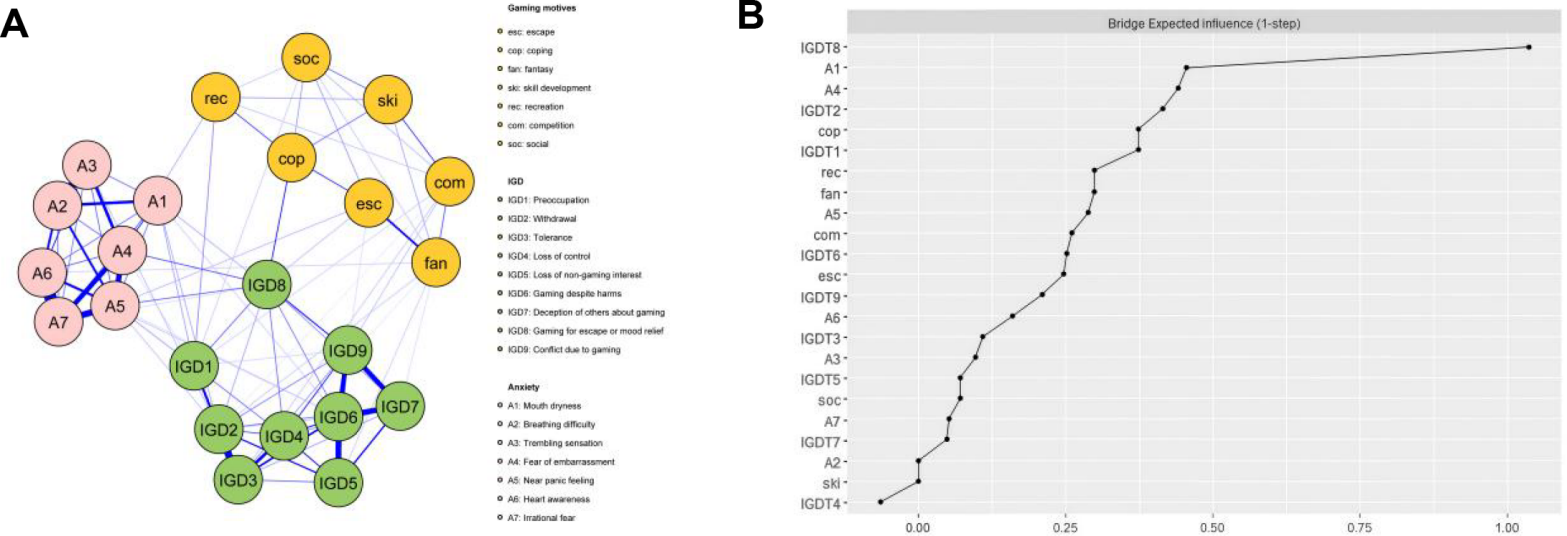
The anxiety-gaming motives-IGD network model and bridge expected influence (BEI). **(A)** The anxiety-gaming motives-IGD network model. **(B)** The bridge expected influence indices of the nodes in the network (raw score).

**Figure 3** shows the anxiety-gaming motives-IGD network (Model 2). There was a maximum of 253 edges across the communities in the network. In the present research, there were 97 cross-community edges(mean weight= 0.095). Bootstrapped confidence intervals(CIs) of the bridge edge weights are shown in **Supplementary Figure 2**.

The bridge expected influence (BEI) values for each node are displayed in **Figure 3B**. Gaming for escape or mood relief (IGD8), Mouth dryness (A1), Fear of embarrassment (A4), Withdrawal(IGD2), and Coping(cop) had the largest 1-step value and emerged as bridge nodes in the combined network model, with BEI values of 1.04, 0.45, 0.44, 0.42 and 0.37, respectively. As **Figure 2B** shows, the average correlation of BEI indices of the subsample and the original sample showed a downward trend as the subsample size decreased. The value of the CS-coefficient on the BEI was 0.75, which was larger than 0.5, indicating adequate stability.

**Figure 4:**
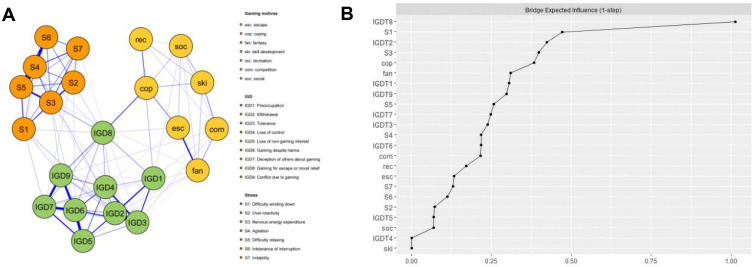
The stress-gaming motives-IGD network model and bridge expected influence (BEI). **(A)** The stress-gaming motives-IGD network model. **(B)** The bridge expected influence indices of the nodes in the network (raw score).

The stress-gaming motives-IGD network (Model3) is displayed in **Figure 4**. There was a maximum of 253 edges across the communities in the network. In the present research, there were 107 cross-community edges (mean weight= 0.096). Bootstrapped confidence intervals (CIs) of the bridge edge weights are shown in **Supplementary Figure 3**.

The bridge expected influence (BEI) values for each node are displayed in **Figure 4B**. Gaming for escape or mood relief (IGD8), Difficulty winding down (S1), Withdrawal (IGD2), Nervous energy expenditure (S3) and coping (cop) had the largest 1-step value and emerged as bridge nodes in the combined network model, with BEI values of 1.01, 0.47, 0.42, 0.40 and 0.38, separately. As **Figure 2C** shows, the average correlation of BEI indices of the subsample and the original sample exhibited a downward trend as the subsample size decreased. The value of the CS-coefficient on the BEI was 0.672, which was larger than 0.5, indicating adequate stability.

## 4 Discussion

The current study used network analysis to examine the association network among psychological distress, gaming motives, and IGD symptoms. Throughout the identification of bridge nodes, we gained a clearer understanding of the key gaming motives and emotional symptoms that influence IGD symptoms, and provided suggestions for IGD interventions through the evaluation of the BEI index.

Within the combined model 1, we identified three key bridge symptoms, which are No initiative (D2), Gaming for escape or mood relief (IGD8) and fantasy motive (fan), and there was a significant positive correlation between any two of them. Our finding aligns with the Compensatory Internet Use Theory (CIUT). According to CIUT (49), individuals use the internet to alleviate negative emotions or escape from real-life problems. Among teenagers, no initiative (D2) often manifests as difficulty in engaging in activities beneficial to physical and mental development, such as learning (50) and exercising (51), which may lead to the situation where individuals gradually find it increasingly difficult to achieve a sense of accomplishment in real life. Besides, adolescents often have a discrepancy between their perception of the real self and the idealized virtual self (52), so they usually hope to step out of their usual identities, try out new identities in fantasy worlds, or attempt things that are impossible in real life. The research results support Davis’ cognitive-behavioral model (53), which indicated that maladaptive cognition is the core of internet addiction. Motive plays a mediating role in the effect of maladaptive cognition on internet addiction (54). Some mediation analyses (55, 56) identified escape motivation as the strongest predictor of IGD severity and a mediator between depression and IGD. While a previous network of gaming motive and IGD found ‘Enjoy being in the gaming world’(fantasy motive) (13) plays a crucial role in the deterioration of IGD symptoms, which was consistent with our research. The Self-discrepancy theory (57) states that the significant disparity between an individual’s actual self and their ideal self is associated with dejection-related emotions (e.g. sadness), which drives them to integrate these self-perceptions, so adolescents may choose internet games as a way to alleviate those emotions. Besides, our study, similar to most studies (58, 59), found Gaming for escape or mood relief (IGD8) is the core bridge node, suggesting that it play a central role in the comorbidities. However, other research also found that preoccupation (IGD1) (13) serves as a key node of increasing the gaming motives, it is possibly due to the close relationship between the fantasy motive and escapism (60, 61), or it could be we also take the relationship between the IGD and depressive symptoms into account.

The anxiety-gaming motives-IGD network model revealed the bridge symptoms were gaming for escape or mood relief (IGD8), Mouth dryness (A1), Fear of embarrassment (A4), withdrawal (IGD2), and coping motive (cop). These results conforms to the I-PACE model. According to the I-PACE model (62), the coping is the most intuitive psychological mechanism for explaining the obstacles to internet use, adolescent with high level of anxiety tend to use the avoidance coping strategy (63) of games to manage interpersonal stress in real life. The fear of embarrassment among adolescents can indirectly reflects the social phobia (64). Previous studies have found that social phobia is a potential key target for intervening in IGD (33), anxiety can promote pathological gaming behavior by affecting social intelligence (65), which is highly consistent with our findings. In face-to-face social interactions, socially anxious individuals may feel stress and uncomfortable, but they can hide themselves in internet gaming environments to find psychological comfort. Therefore, they eventually resorted to indulging in the internet games as a way to escape from reality or to alleviate negative emotions, which is known as IGD8. Besides, mouth dryness is a typical physiological manifestation of excessive activation of the sympathetic nervous system in an anxious state (66). When adolescents are in highly aroused and uncomfortable state, various stimuli in the game may enable them to ignore the internal discomfort signals from the real world.

Within the combined model 3, the bridge nodes were gaming for escape or mood relief (IGD8), difficulty winding down (S1), withdrawal (IGD2), nervous energy expenditure (S3), and coping motive (cop). Our study highlights the specific role of somatic stress symptoms, and these results conforms to the I-PACE model as well. Similarly to mouth dryness, difficulty winding down (S1) and nervous energy expenditure (S3) also reflects physical discomfort. These physiological discomfort urgently requires a release channel, so adolescents increased their coping motive with the above-mentioned physical and psychological discomfort. However, when an individual stops playing the game, the withdrawal reaction (IGD2) is usually accompanied by irritability and anxiety, which highly overlaps with the physical discomfort caused by anxiety and stress (67). To cope with the withdrawal symptoms, individuals reinforce their motive to play games, thus creating a vicious cycle.

While our study revealed the interactive relationship among psychological distress (depression, anxiety, stress), gaming motives, and IGD for the first time. However, the present study still has some limitations. First, the use of cross-sectional data meant that we can only determine the correlation between the research variables but cannot establish the causal relationship between them. Second, our study just focused on the correlation among emotions, motivations and behaviors, without further exploring whether motivations have a mediating or moderating effect in this relationship. In addition, reliance on self-reported data collected through questionnaires may introduce various methodological biases. Third, in order to apply the current network model and causal search algorithm, the current study had to simplify the symptom scores of DASS-21 and IGDT-10 from multi-class ordered data to binary variables (0/1), which was because that the algorithm is still unable to effectively handle highly skewed multi-class data. However, it might result in the loss of some information. Finally, the network analysis performed in this study only examined the relationships between variables at a single point and did not consider the dynamics of these relationships over time.

Future research should utilize longitudinal data to capture temporal fluctuations and interactions establish causal pathways to gain a more complete understanding of the underlying processes by Cross-Lagged Panel Network (CLPN) or Mediation Analysis.

## 5 Conclusion

This study provides a comprehensive network analysis of depressive, anxious, stressful emotions, gaming motives related to IGD and IGD symptoms, suggesting fantasy motive and coping motive as bridges connecting psychological distress to IGD. Besides, the present study identified precise targets for clinical intervention, including no initiative, mouth dryness, fear of embarrassment, difficulty winding down, and nervous energy expenditure, thereby more effectively to reduce the risk of IGD.

## Data Availability

The raw data supporting the conclusions of the article are not publicly available due to the sensitive nature of the data (e.g., participant information). Requests to access the data should be directed to the corresponding author(s).
