# Supplementary material for: Associations of psychological distress, gaming motives and internet gaming disorder in adolescents: a network analysis
Source: Front Psychiatry. 2026 Apr 24;17:1787380. doi: 10.3389/fpsyt.2026.1787380 (PMC13153085; doi:10.3389/fpsyt.2026.1787380)
Supplement: Supplementary file 1 [file SupplementaryFile1.docx]

Supplementary Material

# Supplementary Figures

##
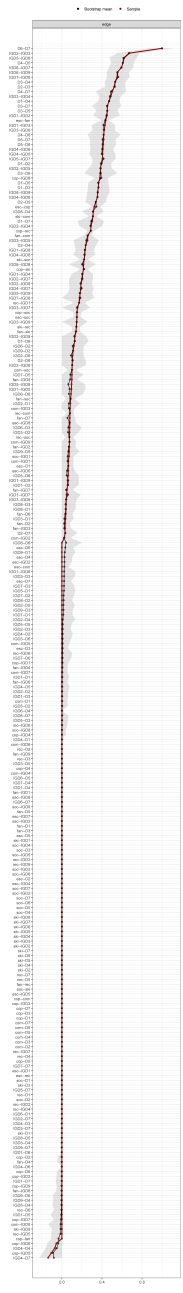


## **Supplementary Figure 1.** Accuracy test of edge weights in the depression-gaming motive-IGD network. The red line indicates the sample edge weight values and the gray area indicates the bootstrapped confidence intervals. Esc, escape; Cop, coping; Fan, fantasy; Ski, skill development; Rec, recreation; Com, competition; Soc, social; IGD1, preoccupation; IGD2, withdrawal; IGD3, tolerance; IGD4, loss of control; IGD5, loss of non-gaming interest; IGD6, gaming despite harms; IGD7, deception of others about gaming; IGD8, gaming for escape or mood relief; IGD9, conflict due to gaming; D1, no positive; D2, no initiative; D3, no look forward; D4, down-hearted; D5, not enthusiastic; D6, worthless; D7, meaningless.

**Supplementary Figure 2.** Accuracy test of edge weights in the anxiety-gaming motive-IGD network. The red line indicates the sample edge weight values and the gray area indicates the bootstrapped confidence intervals. Esc, escape; Cop, coping; Fan, fantasy; Ski, skill development; Rec, recreation; Com, competition; Soc, social; IGD1, preoccupation; IGD2, withdrawal; IGD3, tolerance; IGD4, loss of control; IGD5, loss of non-gaming interest; IGD6, gaming despite harms; IGD7, deception of others about gaming; IGD8, gaming for escape or mood relief; IGD9, conflict due to gaming; A1, mouth dryness; A2, breathing difficulty; A3, trembling sensation; A4, fear of embarrassment; A5, near panic feeling; A6, heart awareness; A7, irrational fear.


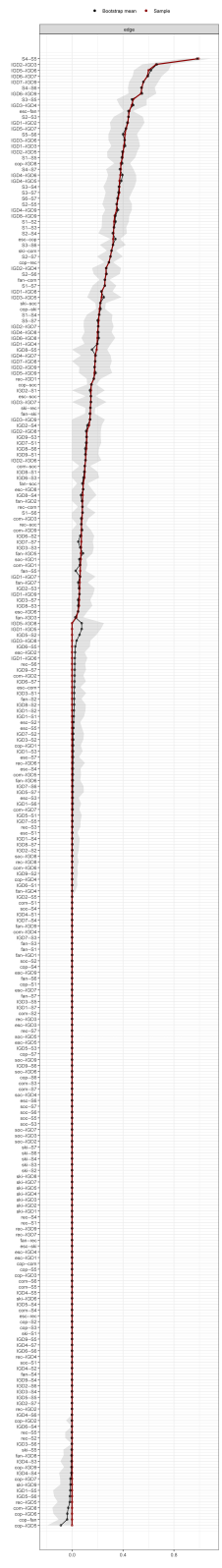


**Supplementary Figure 3.** Accuracy test of edge weights in the stress-gaming motive-IGD network. The red line indicates the sample edge weight values and the gray area indicates the bootstrapped confidence intervals. Esc, escape; Cop, coping; Fan, fantasy; Ski, skill development; Rec, recreation; Com, competition; Soc, social; IGD1, preoccupation; IGD2, withdrawal; IGD3, tolerance; IGD4, loss of control; IGD5, loss of non-gaming interest; IGD6, gaming despite harms; IGD7, deception of others about gaming; IGD8, gaming for escape or mood relief; IGD9, conflict due to gaming; S1, difficulty winding down; S2, over-reactivity; S3, nervous energy expenditure; S4, agitation; S5, difficulty relaxing; S6, intolerance of interruption; S7, irritability.





**Supplementary Figure 4.** Accuracy test of bridge expected influences in the depression-gaming motive-IGD network. The red line indicates the sample edge weight values and the gray area indicates the bootstrapped confidence intervals. Esc, escape; Cop, coping; Fan, fantasy; Ski, skill development; Rec, recreation; Com, competition; Soc, social; IGD1, preoccupation; IGD2, withdrawal; IGD3, tolerance; IGD4, loss of control; IGD5, loss of non-gaming interest; IGD6, gaming despite harms; IGD7, deception of others about gaming; IGD8, gaming for escape or mood relief; IGD9, conflict due to gaming; D1, no positive; D2, no initiative; D3, no look forward; D4, down-hearted; D5, not enthusiastic; D6, worthless; D7, meaningless.





**Supplementary Figure 5.** Accuracy test of bridge expected influences in the anxiety-gaming motive-IGD network. The red line indicates the sample edge weight values and the gray area indicates the bootstrapped confidence intervals. Esc, escape; Cop, coping; Fan, fantasy; Ski, skill development; Rec, recreation; Com, competition; Soc, social; IGD1, preoccupation; IGD2, withdrawal; IGD3, tolerance; IGD4, loss of control; IGD5, loss of non-gaming interest; IGD6, gaming despite harms; IGD7, deception of others about gaming; IGD8, gaming for escape or mood relief; IGD9, conflict due to gaming; A1, mouth dryness; A2, breathing difficulty; A3, trembling sensation; A4, fear of embarrassment; A5, near panic feeling; A6, heart awareness; A7, irrational fear.





**Supplementary Figure 6.** Accuracy test of bridge expected influences in the stress-gaming motive-IGD network. The red line indicates the sample edge weight values and the gray area indicates the bootstrapped confidence intervals. Esc, escape; Cop, coping; Fan, fantasy; Ski, skill development; Rec, recreation; Com, competition; Soc, social; IGD1, preoccupation; IGD2, withdrawal; IGD3, tolerance; IGD4, loss of control; IGD5, loss of non-gaming interest; IGD6, gaming despite harms; IGD7, deception of others about gaming; IGD8, gaming for escape or mood relief; IGD9, conflict due to gaming; S1, difficulty winding down; S2, over-reactivity; S3, nervous energy expenditure; S4, agitation; S5, difficulty relaxing; S6, intolerance of interruption; S7, irritability.
